# Serum mineral concentrations in the patients of surgical intensive care unit

**DOI:** 10.1186/2197-425X-3-S1-A584

**Published:** 2015-10-01

**Authors:** J-M Lee, ES Bang

**Affiliations:** Ajou University School of Medicine, Suwon, Republic of Korea; Ajou University Hospital, Suwon, Republic of Korea

## Introduction

Concentrations of some circulating trace elements such as zinc and selenium are known to decrease significantly after severe trauma, surgery, sepsis, and severe systemic inflammatory response and decreased plasma trace elements concentrations are associated with severity of critical illness.

## Objectives

We examined the value of plasma trace element, zinc (Zn), copper (Cu), manganese (Mn) and selenium (Se) when assessing nutritional status in critically ill patients in the surgical intensive care unit (ICU).

## Methods

A total of 54 patients (34 males and 20 females, mean age of 64 years old) who were admitted to surgical ICU and consulted to the nutritional support team (NST). Arterial blood samples were obtained on the day of NST consultation and every 7 days until discharge from the ICU. The concentrations of trace elements were measured at the Seoul Medical Science Institute (Seoul, Korea). The references of trace elements were like this; Zn (66-110 µg/dl), Cu (75-145 µg/dl), Mn (4.7 - 18.3 µg/l), and Se (5.8-23.4) µg/dl.

## Results

The mean length of stay in the ICU and mortality were 22.7 days (median 14 days) and 24%, respectively. The Zn deficiencies were detected in 72%. The mean concentration of Zn on the day of NST consultation was 55.5 ± 22.9 µg/dl and it recovered to normal range to 89.5 ± 29.3 µg/dl on the following day 14 after TPN and trace elements requirement drug appliance. There was 36% of Cu deficiency, only one case of Se deficiency and no Mn deficiency. Both Cu and Mn showed increased concentration in normal range; Cu : 83.7 ± 22.2 µg/dl à 108.9 ± 32.4 µg/dl and Mn : 9.4 ± 4 µg/l à13.8 ± 3.7µg/l, respectively.

## Conclusions

There was high incidence of Zn deficiency (72.2%). Zn deficiency can induce the impaired wound healing, diarrhea and immunosuppression, therefore, we recommend regular checkup of serum Zn level and strict supplement. Compared to Zn, other trace elements showed quite normal range of serum concentration. Further study about trace elements and nutritional status and clinical outcome in the ICU patients are needed.

